# A novel polysaccharide from *Lentinus edodes* mycelia protects MIN6 cells against high glucose-induced damage via the MAPKs and Nrf2 pathways

**DOI:** 10.29219/fnr.v63.1598

**Published:** 2019-06-06

**Authors:** Xiangyu Cao, Dan Liu, Ying Xia, Tiange Cai, Yin he, Jianli Liu

**Affiliations:** School of life Science, Liaoning University, Shenyang, Liaoning, China

**Keywords:** LMP, MIN6 cells, ROS, Oxidative stress, MAPK, Nrf2

## Abstract

**Background:**

Diabetes mellitus is one of the most widespread diseases in the world, high glucose can damage islet cells, it is important to discover new natural products to inhibit high glucose damage. The protective effects and mechanisms of a novel Lentinus edodes mycelia polysaccharide (LMP) against damage induced by high glucose in MIN6 cells were explored.

**Methods:**

Cell viability, malondialdehyde (MDA) inhibition, lactate dehydrogenase (LDH) release and the activity of superoxide dismutase (SOD) were evaluated under 40 mM glucose with or without LMP for 48 h. Cell signaling pathway analysis was performed to investigate the possible mechanisms of the protective effects of LMP in MIN6 cells.

**Results:**

The results showed that LMP could increase cell viability and the activity of SOD, decrease the reactive oxygen species ( ROS) production, and reduce the MDA content and LDH release in high glucose-induced MIN6 cells. Moreover, LMP prevented high glucose-induced apoptosis by decreasing the expression of Bax and the activation of caspase-1 and caspase-3. Cell signaling pathway analysis showed that p38 mitogen-activated protein kinase (MAPK) and JNK pathways were inhibited and the Nrf2 pathway was activated after treated with LMP.

**Conclusion:**

The protective effects of LMP against MIN6 cells damage induced by high glucose might rely on the regulation of the MAPK and Nrf2 pathways. These results indicated that LMP had great potential as a therapeutic agent for the treatment of diabetes mellitus.

## Popular scientific summary

- Polysaccharide from *Lentinus edodes* mycelia inhibits oxidative stress induced by high glucose in MIN6 cells.
- Polysaccharide from *Lentinus edodes* mycelia inhibits high glucose-induced apoptosis of MIN6 cells.
- Polysaccharide from *Lentinus edodes* mycelia protecting MIN6 cells against high glucose-induced damage may rely on mediating MAPK and Nrf2 pathway.

Type 2 diabetes mellitus (T2DM) is a metabolic disease, which leads to a high risk of complications and high morbidity (1). In 2017, there were 451 million people with diabetes worldwide, and it is expected that this figure will increase to 693 million by 2045 (2). Diabetes refers to a group of lifelong metabolic diseases characterized by chronic hyperglycemia due to insufficient insulin secretion and/or biological dysfunction (3). For T2DM, insulin resistance and pancreatic cell failure are the main factors, with a complex inter-relationship responsible for initiating its pathogenesis (4).

At present, the main clinical drugs for T2DM include sulfonylureas, α-glucosidase inhibitors and biguanides, but these drugs are associated with side effects (5). Active substances derived from natural products are generally considered to be less toxic than chemically synthesized drugs (6). Some natural products, especially the polysaccharides, extracted from medicinal plants and mushrooms have been used in the treatment of diabetes (7, 8). Polysaccharides extracted from mulberry were able to decrease blood glucose in rats (9). The natural biomacromolecule *Dendrobium officinale* polysaccharide had a remarkable hypoglycemic effect in mice with STZ-induced T2DM (10). In fact, several polysaccharide products have been used to treat DM in China, such as konjac glucomannan and pumpkin polysaccharide (11).

*Lentinus edodes* is one of the most popular edible fungi in the world. It is rich in various nutrients and biologically active substances, such as proteins, vitamins, minerals, and polysaccharides (12, 13). The production of fruiting bodies needs a long cultivation in a plastic bag, and its product quality is difficult to control; therefore, mycelia are the alternative or substitute products of mature fruiting bodies, for use in the formulation of nutraceuticals and medicine (14). At present, polysaccharides from mycelia have become a research hotspot in the field of polysaccharides. A new polysaccharide extracted from the *Lentinus edodes* mycelium (LMP) has been prepared in our laboratory, which exhibits antioxidant activity in islet cells (15). However, the inhibitory effects of these polysaccharides against glucose cytotoxicity are still undetermined. In this study, the protective effects of LMP on glucose-induced cell damage in MIN6 cells and the associated mechanism were explored, which might provide a basis for developing natural drugs for diabetes treatment.

## Materials and methods

### Chemicals and reagents

Hochest 33258 staining kit, LDH, MDA and SOD kits were purchased from Jiancheng Biologic Project Company (Nanjing, China); 4% paraformaldehyde and Bax (cat. no. sc-23959), Bcl-2 (cat. no. sc-509), nuclear factor erythroid 2-related factor 2 (Nrf2; cat. no. sc-518033), PCNA (cat. no. sc-25280), Caspase-1 (cat. no. sc-56036), and Caspase-3 (cat.no. sc-271028) were purchased from Santa Cruz Biotechnology, Inc. (Dallas, TX, USA). β-actin (cat. no. 4970S), anti-p38 (cat. no. 9212), anti-phospho-p38 (cat. no. 9215), JNK (cat. no. 9252), and p-JNK (cat. no. 9251) antibodies were purchased from Cell Signaling Technology, Inc. (Danvers, MA, USA). Anti-rabbit IgG (cat. no. SE134) and anti-mouse IgG secondary antibodies (cat. no. SE131) were obtained from Beijing Solarbio Science & Technology Co., Ltd. (Beijing, China).

### Cell culture

MIN6 cells were obtained from the Cell Bank of Type Culture Collection of Chinese Academy of Sciences (Shanghai, China). MIN6 cells were grown in DMEM supplemented with 10% fetal bovine serum, 100 U/mL of penicillin and 100 μg/mL of streptomycin at 37˚C in an incubator with 5% CO_2_.

### MTT assay

MIN6 cells were seeded into 96-well plates at a density of 3 × 10^3^ cells/well. Then, cells were treated with 40 mM glucose and various concentrations (0.0125, 0.025, and 0.05 mM) of LMP for 48 h at 37˚C; 20 μL of 5 mg/mL MTT was added to each well, followed by incubation for 4 h. The medium was removed and 150 μL dimethyl sulfoxide (DMSO) was added to each well to dissolve the thiazolyl blue tetrazolium bromide (MTT) formazan crystals. The optical density value was measured at 490 nm with a microplate reader.

### Hoechst 33258 fluorescence staining

MIN6 cells were seeded in 12-well plates (8 × 10^4^ cells/well) and treated with 40 mM glucose and different concentrations (0.0125, 0.025, and 0.05 mM) of LMP. Cells were washed twice with Buffer A for 5 min. After incubation with 4% paraformaldehyde solution for 30 min, cells were stained with 100 μL Hoechst 33258 for 10 min at room temperature, washed with Buffer A and mounted using 50% glycerol. Cells were observed with fluorescence microscope (20× magnification) (16).

### ROS and LDH determination

The ROS were determinate by 2', 7'-dichlorofluorescein (DCFH-DA) (17). The cells were seeded in 12-well plates at a density of 8 × 10^4^ cells per well and cultured for 48 h. The supernatant was collected from the plates after treatment. Then, cells were incubated with 500 μL dichlorodihydro-fluorescein diacetate (DCFH-DA) for 45 min at 37˚C in dark. Cells were washed twice with phosphate buffered saline (PBS) for 5 min. After incubation with 4% paraformaldehyde solution for 30 min, cells were observed with a fluorescence microscope (10× magnification). The supernatant was collected and the LDH release was determined with LDH assay kit, according to the manufacturer’s instructions. The absorbance was recorded at 450 nm.

### MDA and SOD assay

Cells were treated as described in 2.4. After treatment, the MIN6 cells were washed twice with PBS, repeated freezing, and thawing to obtain homogenate. The homogenate was centrifuged at 4,000 rpm for 15 min, and the supernatant was collected for the MDA and SOD assay using commercial kits. The level of lipid peroxidation was indicated by the amount of MDA in the cells. The MDA and SOD content were determined by using MDA and SOD assay kits, according to the manufacturer’s instructions. The absorbance was recorded at 532 and 550 nm, respectively.

### Western blot assay

The cells were cultured in flasks. When the growth density reached 60%, glucose and different concentrations (0.0125, 0.025, and 0.05 mM) of LMP were added for 48 h. Cells were harvested and lysed, and total protein extracts, cytoplasmic extracts, and nuclear extracts were prepared. The protein concentration of the extracts was estimated with bicinchoninic acid protein assay according to the manufacturer’s instruction. Equal amounts of total cell proteins (20 μg) were loaded and separated by 12% SDS poly-acrylamide gel electrophoresis (SDS-PAGE) and transferred to PVDF membranes by electroblotting. After blocking with 5% non-fat milk for 1 h, the blots were incubated with primary antibodies overnight at 4˚C. Primary antibodies were used at a dilution of 1:2,000. The membranes were washed with TBST and incubated with the secondary antibodies at a dilution of 1:5,000 for 1 h. Finally, chemiluminescent detection was performed by using ECL reagents. β-actin and PCNA were used as loading controls. Densitometry analysis was performed by using the ImageJ software (National Institutes of Health, Bethesda, MD, USA).

### Statistical analysis

Data are presented as the mean ± standard deviation of at least three independently performed experiments. Statistical analysis was conducted by one-way analysis of variance (ANOVA) by using SPSS software version 20.0 (SPSS, Inc., Chicago, IL, USA). *P* < 0.05 and *P* < 0.01 were considered statistically significant.

## Results

### Effect of LMP on cell viability

**Fig. 1:**
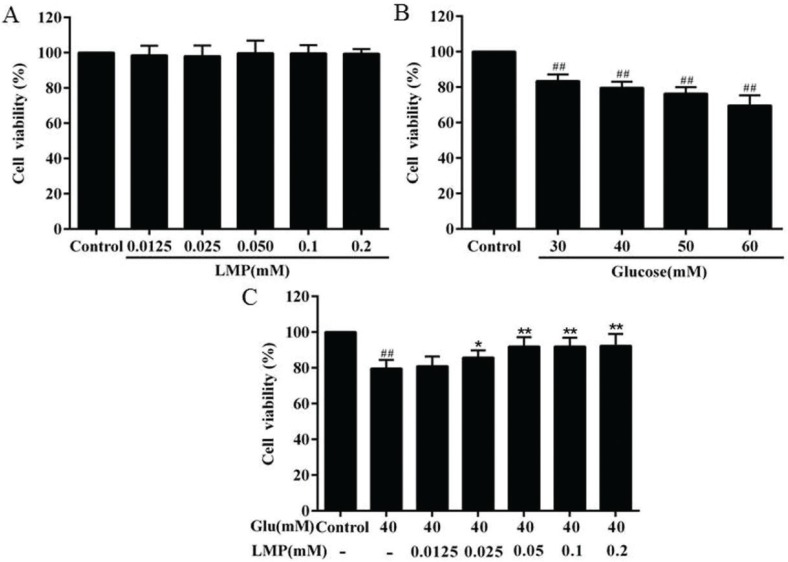
Protective effects of LMP on cell viability losses in MIN6 cells induced by glucose. (A) MIN6 cells were incubated with 0.0125, 0.025, 0.050, 0.1, 0.2 mM LMP for 48 h. Then the cell viability was evaluated by MTT assay. (B) The MIN6 cells were treated with glucose at different concentrations (30, 40, 50 and 60 mM) for 48 h. The cell viability was evaluated by MTT assay. (C) Protective effects of LMP on cell viability losses in MIN6 cells induced by 40 mM glucose. The untreated normal cells (control group) were assigned values of 100% and data are expressed as the mean ± standard deviation. *##P* < 0.01 vs. control group, *P < 0.05, **P < 0.01 vs. group treated with glucose.

Cell viability was quantified by MTT assay. The result revealed that LMP had no significant (*P* > 0.05) effect on cell viability at concentrations between 0.0125 and 0.2 mM (Fig. 1a). The cell viability of MIN6 cells was determined after treatment with different concentrations of glucose. As shown in Fig. 1b, the cell viability of MIN6 cells significantly (*P* < 0.01) decreased in a concentration-dependent manner after treatment with glucose for 48 h, and 40 mM glucose was chosen for the subsequent experiments. As shown in Fig. 1c, after treatment with 0.0125, 0.025, and 0.05 mM LMP for 48 h, the cell viability was restored compared with the glucose-treated group in a concentration-dependent manner.

### Hoechst 33258 fluorescent staining analysis

**Fig. 2:**
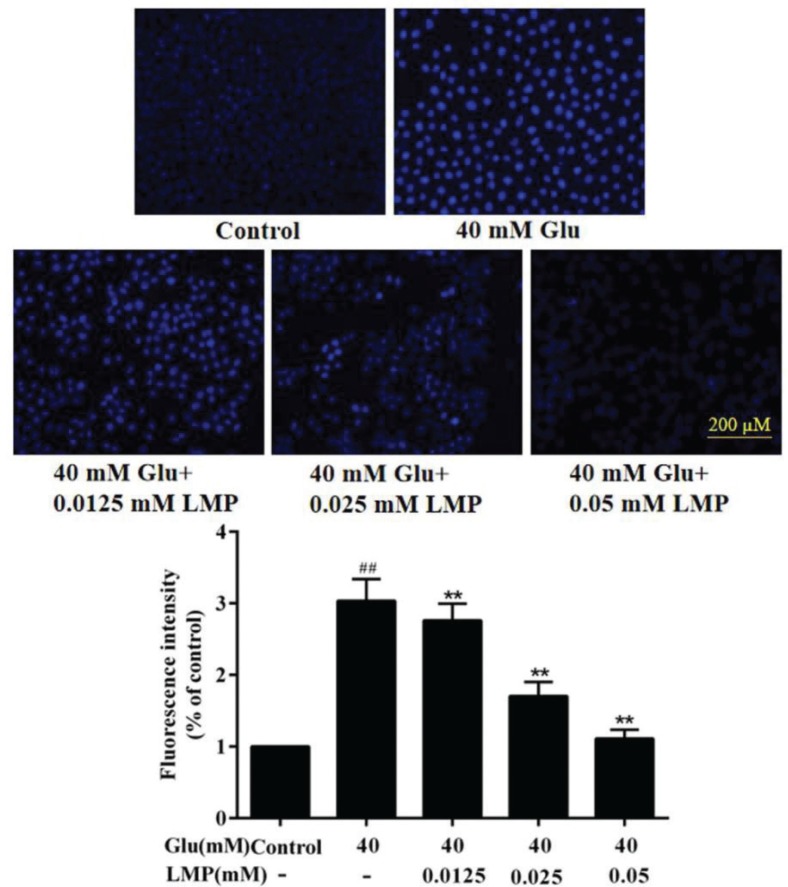
The apoptosis detection of the MIN6 cells. Cells were stained with Hoechst33258 and observed under a fluorescence microscope. Data are expressed as the mean ± standard deviation. *##P* < 0.01 vs. control group, *P < 0.05, **P < 0.01 vs. group treated with glucose.

Hoechst 33258 can permeate the cells and make the nuclei appear blue, which can indicate condensation of chromatin and fragmentation (18). As shown in Fig. 2, after treatment with 40 mM glucose for 48 h, the fluorescence of cells treated with glucose was brighter than that of control cells. When MIN6 cells were incubated with 40 mM glucose and different concentrations of LMP simultaneously, the blue fluorescence gradually decreased with the increasing concentration of LMP. The results showed that LMP could inhibit chromatin fragmentation and condensation induced by glucose in MIN6 cells.

### Effects of LMP on ROS and LDH production

**Fig. 3:**
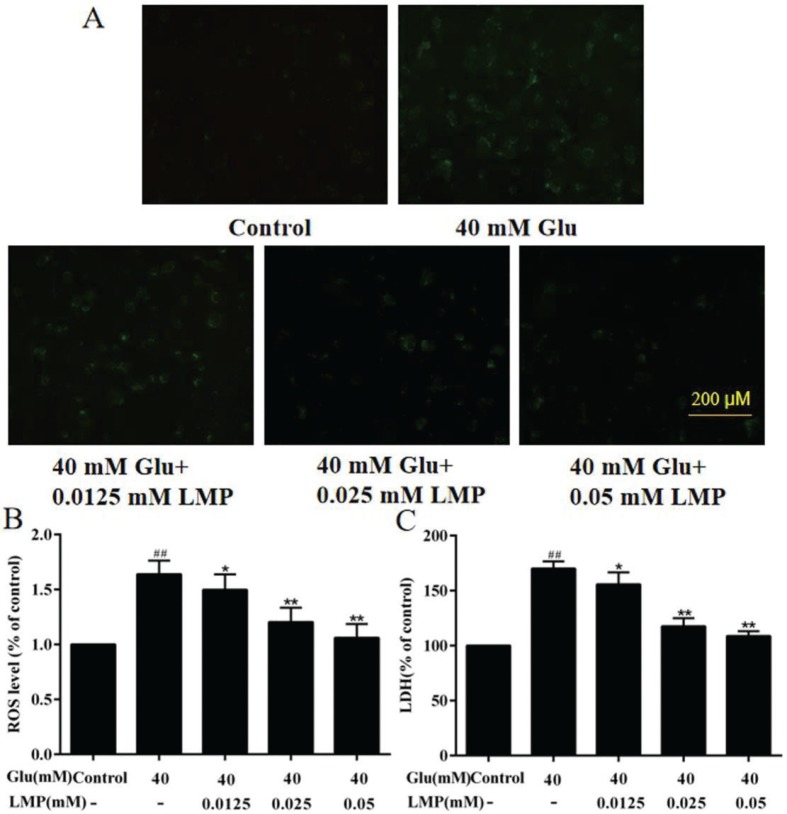
Effects of LMP on ROS release and LDH overproduction in MIN6 cells after exposure to 40 mM glucose. (A) Results of MIN6 cells stained with DCFH-DA. (B) Quantitative analysis of ROS level in MIN6 cells. (C) The LDH release in MIN6 cells. Data are expressed as the mean ± standard deviation. *##P* < 0.01 vs. control group, *P < 0.05, **P < 0.01 vs. group treated with glucose.

As shown in Fig. 3a and b, treatment with 40 mM glucose led to an increase of ROS level in MIN6 cells compared with the control group, and the intracellular ROS levels in MIN6 cells were significantly (*P* < 0.01) reduced when the cells were incubated with 0.025 or 0.05 mM LMP for 48 h. To further investigate the protective effects of LMP in MIN6 cells, the release of LDH was measured. After 40 mM glucose treatment, the release of LDH from MIN6 cells was increased (*P* < 0.01), and LMP reversed this effect significantly (*P* < 0.05). This result indicated that the protective effect of LMP on MIN6 might be related to the inhibition of the release of LDH. Therefore, LMP not only decreased the LDH release, but also reduced ROS production, and thus alleviated glucose-induced cell toxicity in MIN6 cells.

### Effects of LMP on MDA and SOD production

**Fig. 4:**
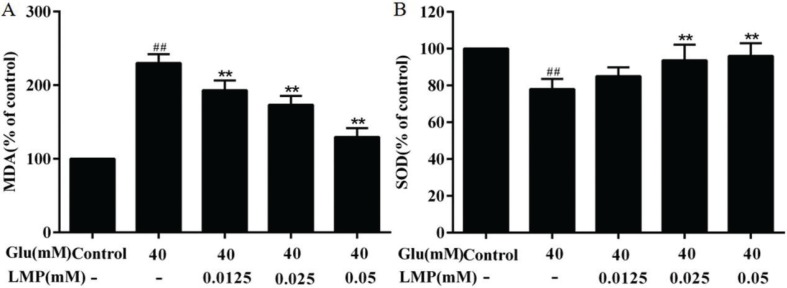
Effects of LMP on the activities of MDA and SOD in MIN6 cells induced by 40 mM glucose. (A) Inhibition of LMP on MDA content in MIN6 cells induced by 40 mM glucose. (B) Promotion of SOD activity in MIN6 cells by LMP after exposure to 40 mM glucose. Data are expressed as the mean ± standard deviation. *##P* < 0.01 vs. control group, *P < 0.05, **P < 0.01 vs. group treated with glucose.

As shown in Fig. 4a, after 40 mM glucose treatment, the level of MDA in MIN6 cells increased significantly (*P* < 0.01), indicating that the lipid peroxidation was enhanced in MIN6 cells. After treated with 0.0125, 0.025, and 0.05 mM LMP and 40 mM glucose together for 48 h, the MDA level in MIN6 cells decreased significantly (*P* < 0.01) with the increasing in LMP concentration, compared with the group treated only with 40 mM glucose. SOD is a scavenger of superoxide anion radicals, as shown in Fig. 4b, there was a significant (*P* < 0.01) reduction of the activity of SOD in the glucose-treated group compared with control group. After LMP was added at the same time, the activity of SOD in MIN6 cells increased gradually with the increase of LMP concentration.

### The expression of apoptosis-related proteins

**Fig. 5:**
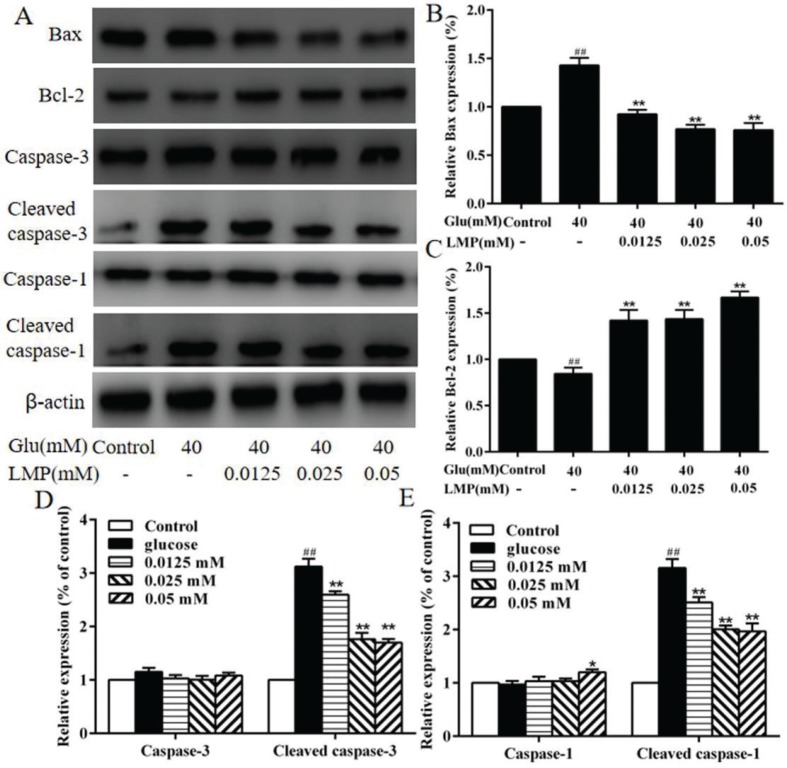
The effects of LMP on the expression of apoptosis-related proteins in MIN6 cells (A) The expression levels of Bax, Bcl-2, Caspase-3, Cleaved caspase-3, Caspase-1 and Cleaved caspase-1 were detected by western blot assay. The levels of β-actin were used as an internal control. (B, C, D and E) Quantitative analysis of the protein expression of Bax, Bcl-2, Caspase-3, Cleaved caspase-3, Caspase-1, Cleaved caspase-1. Data are expressed as the mean ± standard deviation. *##P* < 0.01 vs. control group, *P < 0.05, **P < 0.01 vs. group treated with glucose.

The expression of Bax, Bcl-2, caspase-3, cleaved caspase-3, caspase-1, and cleaved caspase-1 in MIN6 cells was detected by western blot. As shown in Fig. 5, the protein expression of Bcl-2 was decreased significantly (*P* < 0.01) with 40 mM glucose, and the expression of Bax, cleaved caspase-3, and cleaved caspase-1 was increased (*P* < 0.01) after treated with 40 mM glucose compared with control cells, and the addition of LMP could reverse these effects significantly. Meanwhile, glucose and different concentrations of LMP had no obvious (*P* > 0.05) effect on the expression of caspase-3 and caspase-1, respectively (Fig. 5d and e).

### Effect of LMP on the MAPK and Nrf2 pathways

**Fig. 6:**
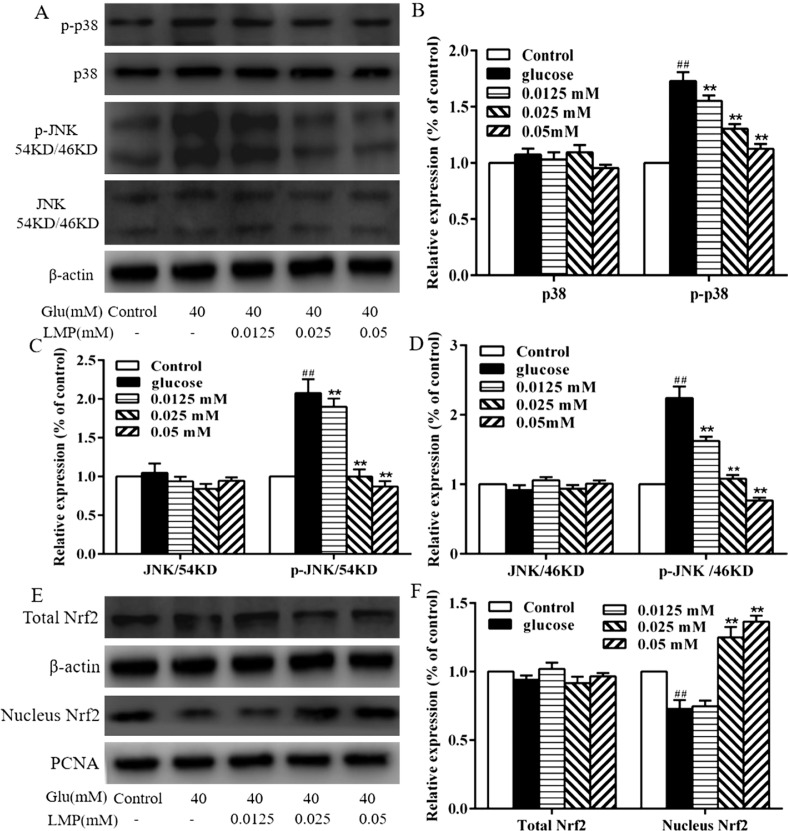
The effect of LMP on MAPK and Nrf2 pathways. (A, B, C and D) The protein expression of p-p38, p38, p-JNK and JNK by western blot analysis using specific antibodies, The levels of β-actin were used as an internal control. (E and F) The expressions of total Nrf2 and Nucleus Nrf2 by western blot analysis using specific antibodies, the levels of β-actin and PCNA were used as an internal control. Data are expressed as the mean ± standard deviation. *##P* < 0.01 vs. control group, *P < 0.05, **P < 0.01 vs. group treated with glucose.

Western blot analysis was used to investigate the effect of LMP on p38 MAPK and JNK expression. As shown in Fig. 6, the expression of total p38 MAPK and JNK did not differ from each group. p-p38 and p-JNK were significantly (*P* < 0.01) increased in 40 mM glucose-treated group compared with the control group. Treatment with LMP led to a decrease in p-p38 and p-JNK in MIN6 cells to basal level (Fig. 6b, c and d). A concentration of 40 mM glucose significantly (*P* < 0.01) decreased the protein expression of Nrf2 in the nucleus, whereas 0.025 and 0.05 mM LMP significantly (*P* < 0.01) increased the translocation of Nrf2 in the nucleus of MIN6 cells. These results indicated that the protective effects of LMP in MIN6 cells damaged by 40 mM glucose might rely on the MAPK and Nrf2 cell-signaling pathways.

## Discussion

Under normal physiological conditions, the production and elimination of ROS in the body is in a dynamic equilibrium (19). Oxidative stress refers to elevated intracellular levels of ROS that cause lipids, protein, and DNA damage, which have been shown to have direct and deleterious consequences in diabetes (20, 21). The study showed that hyperglycemia could generate ROS, which in turn causes oxidative stress on the beta cell (22, 23). Our results showed that high glucose treatment alone significantly increased ROS levels in MIN6 cells, and the level of ROS reduced after treatment with LMP.

Lipid peroxides are the product of the reaction of ROS with polyunsaturated fatty acids, which can reflect the degree of damage to the body caused by oxidative stress (24). MDA, one of the lipid peroxidation products, is regarded as biomarkers of oxidative stress in cells (25). Several previous studies have shown that the level of MDA in subjects with T2DM is increased (26, 27). In this study, we found that MDA level in the group treated with 40 mM glucose was significantly higher than that in the control group, and LMP could decrease the MDA levels compared to the 40 mM glucose-treated group. It has been previously reported that diabetic patients exhibited high ROS production accompanied by decreased expression of antioxidant enzymes (28). SOD, one of the most important antioxidant enzymes, catalyzes the dismutation of O_2_^−^ to H_2_O_2_ and O_2_ and inhibits the formation of free radical (29). It has been reported that pumpkin polysaccharides protected islets cells from STZ injury in vitro via increasing the levels of SOD and reducing the level of MDA (30). The results of the present study showed that the 40 mM glucose significantly reduced the expression of SOD in MIN6 cells; however, SOD activity in MIN6 cells was recovered after treatment with LMP.

Apoptosis, a process of programmed cell death characterized by cell shrinkage, chromatin condensation and DNA cleavage (31). Islet β-cell apoptosis plays an crucial role in the development of T2DM (32, 33). β-cell apoptosis can occur through many pathways (34). Mitochondrial apoptotic pathway can be regulated by Bcl-2 family and caspase family (32). It has been reported that polysaccharide could inhibit pancreatic β-cells apoptosis through regulating the expression of Bcl-2 family and caspase family proteins (35, 36). Our study showed that LMP could inhibit MIN6 cells apoptosis by regulating mitochondrial apoptotic pathway.

MAPK is an important signaling pathway and plays a key role in regulating cellular function (37). MAPK cascade is the divergent combination of at least three protein kinases such as MAPKKK (MKKK/MEKK, MAP3K), MAPKK (MEK/MKK), and MAPK (MPK), stimulating each other by phosphorylation (38, 39). ROS induces phosphorylation of MAPK-signaling proteins such as RTK and MAP3K, resulting in the activation of MAPK pathways (40, 41). In eukaryotes, the MAPK-signaling pathways include p38, JNK, and ERK. p38 MAPK- and JNK-signaling pathways are associated with the response of cells to stresses, such as inflammation and ROS (42). Therefore, the inhibition of MAPK-signaling pathway has protective effects against islet cell dysfunction. In our study, glucose activated the p38 MAPK and JNK pathways in MIN6 cells, and LMP decreased the phosphorylation of p38 and JNK in a concentration-dependent manner, indicating that the protective effects of LMP on damage in MIN6 cells induced by glucose might partially be mediated by regulating the MAPK pathway.

The transcription factor Nrf2 is a key protein in the regulation of the endogenous antioxidant response and is considered as a promising therapeutic target for diseases caused by oxidative stress (43). By activating the Nrf2 antioxidant-signaling pathway, the ability of the body to resist oxidative stress damage can be significantly enhanced (44, 45). Therefore, the Nrf2 pathway is a potential target for the treatment of diabetes (46). Nrf2 is a key transcription factor that determines redox status by regulating numerous antioxidant enzymes (47). It has been reported that antrodia cinnamomea polysaccharide (48) and Lycium barbarum polysaccharides could activate Nrf2 (49). It has also been reported that curcumin resulted in enhanced nuclear translocation of Nrf2, which plays a role in the cellular protection against oxidative stress (50). Nrf2 is involved in the expression of various antioxidant proteins (such as detoxifying enzymes) via antioxidant response element binding site, hyperglycemia leads to oxidative stress and results in changes in levels of Nrf2, persistent hyperglycemia decreases its expression of Nrf2, and evidence has also indicated decreased levels of Nrf2 in diabetes (51). In this study, LMP was able to promote nuclear translocation of Nrf2, and the expression of SOD, a downstream enzyme of Nrf2, increased after treatment with LMP.

## Conclusions

The present study showed that LMP could recover the glucose-induced redox changes. These results indicated that LMP might become a potential therapeutic agent for diabetes, due to its inhibition of glucose toxicity.
